# Does the Organ Matter in PTLD Development in Solid Organ Transplant Recipients? A Multicenter Observational Study of Risk and Prognostic Factors

**DOI:** 10.3390/cancers17111770

**Published:** 2025-05-25

**Authors:** Rafał Staros, Bartosz Foroncewicz, Dorota Kamińska, Dominika Dęborska-Materkowska, Sławomir Lizakowski, Izabela Zakrocka, Joanna Raszeja-Wyszomirska, Anita Stanjek-Cichoracka, Anna Pawłowska, Emilia Knioła, Paweł Poznański, Jolanta Gozdowska, Alicja Dębska-Ślizień, Wojciech Załuska, Marek Ochman, Agnieszka Kołkowska-Leśniak, Michał Grąt, Tomasz Stompór, Magdalena Durlik, Radosław Zagożdżon, Zbigniew Gałązka, Maciej Kosieradzki, Krzysztof Zieniewicz, Leszek Pączek, Krzysztof Mucha

**Affiliations:** 1 Department of Transplantology, Immunology, Nephrology and Internal Diseases, Medical University of Warsaw, 02-006 Warsaw, Poland; 2 Division of Nephrology, Transplantology, and Clinical Immunology at the 4th Military Clinical Hospital, Faculty of Medicine, Wroclaw University of Science and Technology, 53-114 Wrocław, Poland; 3 Department of Nephrology, Transplantology and Internal Medicine, Medical University of Gdańsk, 80-210 Gdańsk, Poland; 4 Department of Nephrology, Medical University of Lublin, 20-090 Lublin, Poland; 5 Department of Hepatology, Transplantology and Internal Medicine, Medical University of Warsaw, 02-097 Warsaw, Poland; 6 Department of Biophysics, Faculty of Pharmaceutical Sciences in Sosnowiec, Medical University of Silesia in Katowice, 41-200 Sosnowiec, Poland; 7 Department of Nephrology, Hypertension and Internal Medicine, School of Medicine, Collegium Medicum, University of Warmia and Mazury, 10-561 Olsztyn, Poland; 8 Department of Cardiac, Vascular and Endovascular Surgery and Transplantology, Medical University of Silesia, Silesian Centre for Heart Diseases, 41-800 Zabrze, Poland; 9 Department of Hematology, Institute of Hematology and Transfusion Medicine, 00-791 Warsaw, Poland; 10 Department of General, Transplant and Liver Surgery, Medical University of Warsaw, 02-097 Warsaw, Poland; 11 Laboratory of Cellular and Genetic Therapies, Medical University of Warsaw, 02-097 Warsaw, Poland; 12 Department of General, Vascular, Endocrine & Transplant Surgery, Medical University of Warsaw, 02-097 Warsaw, Poland; 13 Department of General and Transplantation Surgery, Medical University of Warsaw, 02-006 Warsaw, Poland; 14 Department of Clinical Immunology, Medical University of Warsaw, 02-097 Warsaw, Poland; 15 Institute of Biochemistry and Biophysics, Polish Academy of Sciences, 02-106 Warsaw, Poland

**Keywords:** post-transplantation lymphoproliferative disorder, lymphoma, kidney transplantation, liver transplantation, lung transplantation

## Abstract

Background/Objectives: The risk of post-transplantation lymphoproliferative disorder (PTLD) varies according to the type of transplanted organ. To investigate the factors contributing to PTLD development and treatment outcomes, we established a multicenter registry that included patients diagnosed with PTLD within a population of 13,263 kidney, liver, and lung transplant recipients (KTRs, LTRs, and LngTRs, respectively), observed in a period between 2000 and 2023. Methods: The chi-squared test was used to analyze differences in group composition. Univariate and multivariate Cox regression were applied to determine the impact of factors upon PTLD onset and patient survival. Results: Our registry included 58 out of 9432 KTRs, 40 out of 3500 LTRs, and 5 out of 331 LngTRs. The median time to PTLD onset was significantly longer among KTRs (117 months post-transplant) than among LTRs (49 months, *p* < 0.001) and LngTRs (5 months, *p* < 0.001). LTRs treated with tacrolimus developed PTLD later compared to LTRs treated with cyclosporin (*p* = 0.042). In multivariate analysis, older age at first transplantation correlated with earlier disease development in SOTRs (HR = 1.03, *p* = 0.006) and KTRs (HR = 1.04, *p* = 0.003). Older age at first transplantation was also associated with worse survival among KTRs (*p* = 0.045). Conclusions: We identified clear differences in the factors affecting PTLD onset and survival between KTRs and LTRs. Organ-specific analyses are needed to improve our understanding of PTLD risk factors, treatment choices, and clinical outcomes.

## Simple Summary

Post-transplantation lymphoproliferative disorder is considered one of the most life-threatening complications of solid organ transplantation (SOT). Evidence suggests that PTLD risk is influenced by organ graft type. Since only a limited population is at risk of PTLD, the majority of research has been based on either small cohorts of single graft-type recipients or larger cohorts of multiple types of SOT recipients. The conclusions from such studies may not be sufficiently generalizable or may miss organ-specific risk and prognostic factors. To address these limitations, we created a multicenter registry that included 103 PTLD patients diagnosed out of 9432 kidney, 3500 liver, and 331 lung transplant recipients. We used this registry to analyze various risk and prognostic factors according to organ graft types. We found that the constellation of factors affecting the time of PTLD onset and patient survival differs between kidney and liver transplant recipients.

## 1. Introduction

Since Penn et al. first described a series of malignant lymphoma cases among kidney transplant recipients, it has become increasingly apparent that lymphoid neoplasms pose a non-negligible threat to allograft recipients [1]. Additional reported cases, including some that are polyclonal, have led to the recognition of post-transplantation lymphoproliferative disorder (PTLD) as a new disease entity. In the 2022 revision of the World Health Organization classification of lymphoid neoplasms, PTLD is defined as lymphoid proliferations and lymphomas arising in the setting of immune deficiency/dysregulation [2]. Immunodeficiency—or more specifically immunosuppression—in the setting of organ transplant reportedly increases the risk of non-Hodgkin and Hodgkin lymphoma by 10-fold and 4-fold, respectively, among organ graft recipients compared to the general population [3].

The most well-established explanation of PTLD pathogenesis involves Epstein–Barr virus (EBV) replication in an allograft recipient, where suppressed and/or depleted T-cells cannot contain the virus-driven B-cell proliferation and oncogenesis. This scenario accounts for the majority of early-onset cases (<12 months post-transplantation) [4] and is most frequent in pediatric graft recipients who carry the highest risk of a primary EBV infection [5]. PTLD shows a second incidence peak at approximately 10–15 years post-transplantation [6], which exhibits different pathogenic mechanisms since up to 50% of cases are EBV-negative [7], and the disease stems from T/NK cells in up to 10% of cases [8,9].

The available evidence suggests that PTLD risk is influenced by organ graft type, EBV donor/recipient mismatch, and the cumulative dose or duration and intensity of immunosuppression (including induction and rejection treatment), while the impact of other factors remains ambiguous [10]. Multiorgan and intestinal graft recipients exhibit the highest relative risk (RR = 239.5), followed by lung transplant recipients (LngTRs, RR = 58.6), pancreas transplant recipients (RR = 34.9), liver transplant recipients (LTRs) (RR = 29.9), heart transplant recipients (RR = 27.6), and kidney transplant recipients (KTRs) (RR = 12.6) [11,12].

Since only a limited population is at risk of PTLD, the majority of research focusing on risk and prognostic factors has been based on either small cohorts with a single graft type [13,14,15,16] or larger cohorts of solid organ transplant recipients (SOTRs) and hematopoietic stem cell transplant (HSCT) recipients [17,18,19,20]. Notably, these approaches may yield biased results. The conclusions from single-center studies may not be sufficiently generalizable to other transplant cohorts, while conclusions from studies that pool data across multiple types of SOTRs may lack granularity and miss organ-specific risk and prognostic factors.

To address these limitations, we created a multicenter registry of PTLD among SOTRs. In the present study, we used this registry to analyze various risk and prognostic factors according to organ graft types.

## 2. Patients and Methods

### 2.1. Study Participants

For this study, we identified patients diagnosed with PTLD from the six transplantation centers participating in this project: Department of Transplantology, Immunology, Nephrology, and Internal Diseases, Medical University of Warsaw (KTRs and LTRs); Department of Nephrology and Transplantation Medicine, Wroclaw Medical University (KTRs); Department of Nephrology, Transplantology, and Internal Medicine, Medical University of Gdańsk (KTRs); Department of Nephrology, Medical University of Lublin (KTRs); Department of Hepatology, Transplantology, and Internal Medicine, Medical University of Warsaw (LTRs); Department of Cardiac, Vascular, and Endovascular Surgery and Transplantology, Medical University of Silesia, Silesian Center for Heart Diseases (LngTRs). Each center received the same forms and data collection instructions. As the participating transplantation centers focus on adult patients, we included predominantly transplant recipients who were diagnosed with PTLD as adults (although no age criterion was set) in a time period between 2000 and 2023 with histologically confirmed PTLD. We have excluded hematopoietic stem cell transplant recipients, simultaneous kidney and liver transplant recipients, patients with a history of lymphoma prior to SOT, and patients without a histological confirmation of PTLD diagnosis. The analyzed data were collected from the patients’ medical records, and no central medical databases were used. The collected data included the following: patients’ sex, age at first transplantation, dates and types of SOT, use and type of induction IS therapy, maintenance IS regimen at the time of PTLD diagnosis, number of acute rejection (AR) episodes, type of AR treatment, date of PTLD diagnosis, virological status at PTLD diagnosis (for CMV, EBV, HBV, and HCV), PTLD histology, location of lesions, PTLD treatment types, and outcomes.

The included patients were classified into three groups: KTRs, LTRs, and LngTRs. The KTR group was defined as KTRs, as well as patients who received non-simultaneous kidney and liver transplants with the kidney graft first. The LTR group was defined analogously.

The identified PTLD cases were classified into PTLD subtypes according to the 2022 International Consensus Classification (ICC) of mature lymphoid neoplasms [21]. Among posthumously confirmed PTLD cases, the date of PTLD diagnosis was defined as the date of the first cytological or imaging results suggestive of PTLD. EBV positivity was classified as either active EBV replication, the presence of latent membrane protein 1, or EBV-encoded RNA in situ hybridization (EBER ISH) in histopathological samples.

The design of this study was approved by the bioethics committee of the Medical University of Warsaw (approval no. KB/75/2023).

### 2.2. Statistical Analyses

The chi-squared test was used to analyze statistical differences in clinical characteristics. Univariate and Multivariate Cox regression were applied to assess the effects of variables on the time of PTLD onset and patient survival. Data analysis was performed using R version 3.2.3. *p*-values of <0.05 were considered statistically significant.

## 3. Results

### 3.1. Clinical Characteristics

Among the patients of the six transplantation centers participating in this project, we identified 103 patients diagnosed with PTLD, including 58 KTRs, 40 LTRs, and 5 LngTRs. Based on the data collected from the participating centers and in case of their unavailability, the data from the annual POLTRANSPLANT Bulletin (years 2001–2024) [22], the screened population was estimated to include 9432 KTRs, 3500 LTRs, and 331 LngTRs, (estimated total n = 13,263 patients) resulting in an estimated PTLD incidence of 0.6% among KTRs, 1.1% among LTRs, and 1.51% among LngTRs. The earliest year of first transplantation was 1987, and the earliest year of PLTD diagnosis was 1999 (one patient, diagnosed in December); the latest were 2021 and 2023, respectively. The KTR and LTR groups each included one case of non-simultaneous KTR and LTR. The LngTR group included one pediatric patient. Six KTRs, one LTR, and one LngTR underwent transplantation as minors. Among these patients, two KTRs and one LngTR developed PTLD before reaching adulthood.

The groups did not significantly differ in sex distribution. Three KTRs, three LTRs, and one LngTR underwent re-transplantation prior to PTLD diagnosis. Compared to the other groups, the LTR group had a significantly higher median age at first transplantation (45 years, *p* = 0.009) and median age at PTLD diagnosis (50 years, *p* = 0.005).

With regards to immunosuppressive (IS) treatment, our analysis revealed that KTRs exhibited significantly more frequent use of triple-drug regimens (84.5%, *p* < 0.001), cyclosporin (CsA) (60.3%, *p* < 0.001), and mycophenolate (63.8%, *p* = 0.022). Compared to the other groups, LTRs more commonly used monotherapy (17.5%, *p* = 0.003) and tacrolimus (TAC) (95%, *p* = 0.001), while LngTRs more commonly used sirolimus (40%, *p* = 0.001). Anti-CD25 induction was most frequent among LTRs (60%, *p* < 0.001), and anti-thymocyte globulin (ATG) induction was used only among KTRs. One-third of KTRs and LTRs and one-fifth of LngTRs had a history of glucocorticoid-treated (GCS) AR episodes prior to PTLD diagnosis.

Data regarding prior AR episodes were missing for nine KTRs and four LTRs. Data regarding viral infections were missing or insufficient for a number of KTRs and LTRs. Among the patients with sufficient data, EBV infection was confirmed in 59.4% of LTRs, 45.2% of KTRs, and 80% of LngTRs. Cytomegalovirus (CMV) infection was significantly more frequent in LngTRs (80%, *p* < 0.001). Table 1 summarizes the patient’s clinical characteristics, IS, and viral infections.

### 3.2. Factors Associated with PTLD Development

**Figure 1:**
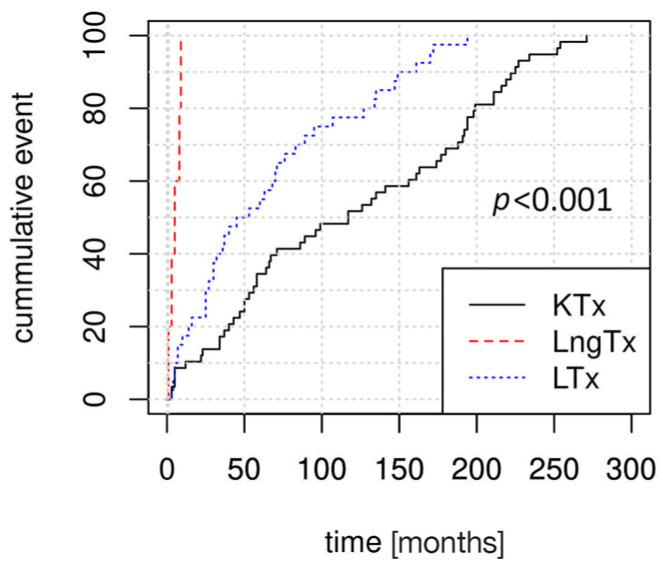
Kaplan–Meier estimator curves depicting differences in the time of PTLD onset between kidney transplant recipients (KTRs, n = 58), liver transplant recipients (LTRs, n = 40), and lung transplant recipients (LngTRs, n = 5). *p*-value for Kruskal–Wallis test comparison of median time to PTLD diagnosis. PTLD, post-transplantation lymphoproliferative disorder.

Kaplan–Meier curves revealed significant differences in the dynamics of PTLD development, according to the type of transplanted organ (Figure 1). PTLD was diagnosed earliest after transplantation in LngTRs (median time to diagnosis: 5 months, IQR: 3–8 months), followed by in LTRs (median: 49 months, IQR: 25–98 months) and in KTRs (median: 117 months, IQR: 50–193.5 months).

**Figure 2:**
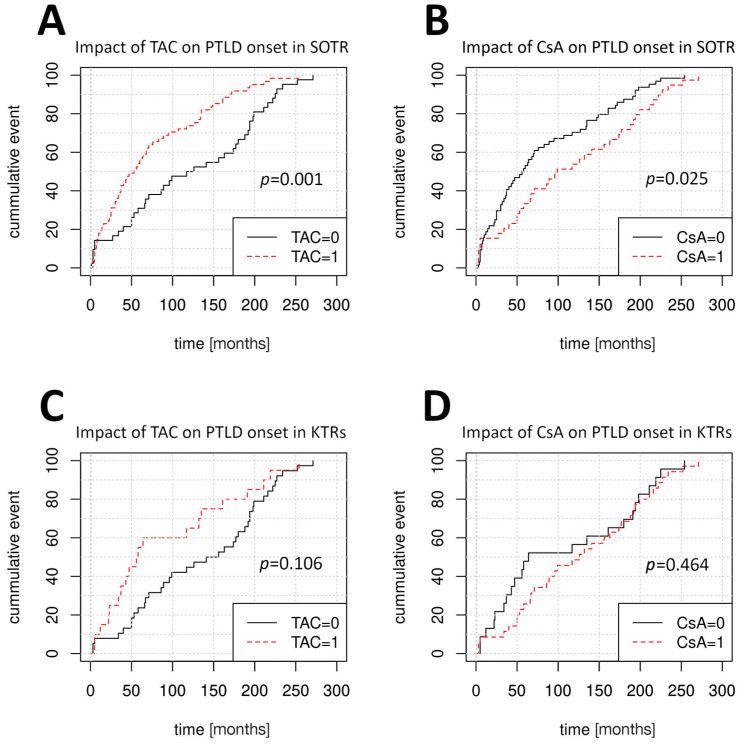

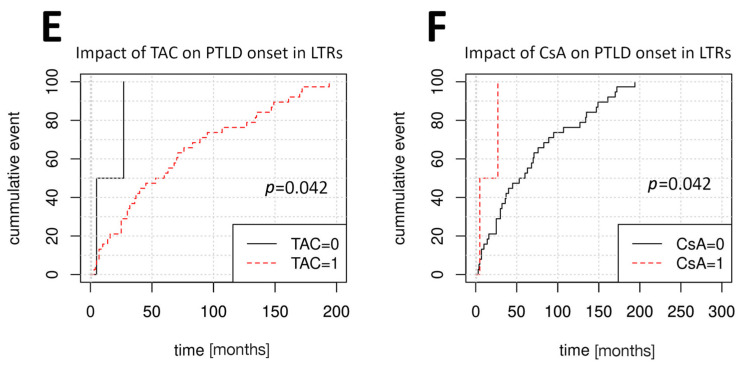
Variable impact of calcineurin inhibitors (CNIs) on the time of PTLD onset. (**A**) Effect of TAC in SOTRs. (**B**) Effect of CsA in SOTRs. (**C**) Effect of TAC in KTRs. (**D**) Effect of CsA in KTRs. (**E**) Effect of TAC in LTRs. (**F**) Effect of CsA in LTRs. *p*-values for univariate Cox regression. PTLD, post-transplantation lymphoproliferative disorder; TAC, tacrolimus; SOTRs, solid organ transplant recipients; CsA, cyclosporine; KTRs, kidney transplant recipients; LTRs, liver transplant recipients.

Univariate Cox regression was performed to identify factors affecting the time of PTLD onset (Table 2). The LngTR cohort size was insufficient for informative analysis. In the pooled SOTR group, disease onset was later among patients who received CsA-based IS regimens (median time to PTLD onset 99 months vs. 57 months for non-CsA regimens, HR = 0.63, 95% CI: 0.42–0.94, *p* = 0.025) and was earlier in older patients (HR = 1.02, 95% CI: 1.00–1.04, *p* = 0.01) and in patients receiving TAC-based (HR = 1.96, 95% CI: 1.3–2.95, *p* = 0.001) or sirolimus-based IS regimens (*p* < 0.001). LTRs developed PTLD later following treatment with TAC (HR = 0.21, 95% CI: 0.05–0.95, *p* = 0.042) compared to treatment with CsA (HR = 4.8, 95% CI: 1.06–21.78, *p* = 0.042). Among KTRs, older age was the only factor that significantly influenced PTLD onset (HR = 1.03, 95% CI: 1.00–1.05, *p* = 0.01). Figure 2 depicts the variable impacts of TAC and CsA treatment at the time of PTLD onset among SOTRs, KTRs, and LTRs. Multivariate Cox regression was used to further verify these findings (Table 3) and determined the patients’ age at first transplantation to be the sole factor affecting PTLD onset among SOTRs and KTRs.

### 3.3. PTLD Type

Early-onset PTLD (<12 months after first transplantation) was diagnosed in 100% of LngTRs, while late-onset PTLD was predominant among LTRs (82.5%) and KTRs (91.4%). Disease dissemination (Lugano stage I vs. Lugano stages II-IV) did not significantly differ between groups. Among the main PTLD subtypes, plasmacytic hyperplasia (PH) PTLD was significantly more common in LTRs (15%) compared to the other groups (*p* = 0.031) (Table 4).

### 3.4. Treatment and Outcomes

The groups did not significantly differ in PTLD treatment, aside from the R-CHOP immuno-chemotherapy regimen, which was most frequently used in LTRs (41%, *p* = 0.01). Among the 30 patients treated with anti-CD20 monotherapy, only seven did not receive subsequent R-CHOP or other chemotherapy regimens.

The survival rate was significantly lower in the KTR group (29.3%, *p* = 0.003), compared to the LTR group (60%) and the LngTR group (80%) (Figure 2). PTLD-related deaths were twice as frequent in the KTR group (43%) compared to in the other groups (*p* = 0.024). The groups did not significantly differ in the frequencies of complete remission and loss to follow-up (Table 5).

### 3.5. Factors Affecting Patient Survival

In the combined cohort of PTLD patients, Lugano stages II-IV and prior ATG administration, as either induction or acute rejection (AR) treatment, were found to be detrimental to patient survival. Among the PTLD treatment modalities, surgery, anti-CD20, and R-CHOP were associated with improved SOTR survival. We reevaluated the impacts of anti-CD20 and R-CHOP among patients with either polymorphic or monomorphic B-cell PTLD only and found the effects to be insignificant in organ-specific groups.

The negative effects of more advanced stages of PTLD (Lugano II-IV) and ATG induction were present in the KTR group. Additionally, immunosuppression reduction was found to be an effective treatment strategy among KTRs (HR = 0.34, 95% CI: 0.14–0.82, *p* = 0.017). In the LTR group, the survival rate was lower among patients with PH-PTLD and those with a history of ATG-treated AR (Table 6). The LngTR cohort was too small for an informative analysis.

We performed multivariate analysis to further validate our findings and found that among SOTRs and KTRs, the main factor affecting survival was patients’ age at first transplantation, factors identified using univariate analysis were found to be insignificant. Among LTRs, plasmacytic hyperplasia PTLD was an independent negative prognostic factor. The results for the multivariate analysis are presented in Table 7.

## 4. Discussion

Using a uniform set of registered data, we verified that the type of transplanted organ affected the frequency of PTLD among SOTRs, in accordance with previous findings. Moreover, we demonstrated that the transplanted organ type influenced a constellation of other factors, impacting the disease dynamics and treatment outcomes. These findings strengthen the evidence against pooling SOTRs for epidemiological analyses of PTLD, as this is a possible source of bias.

Based on their age at transplantation, male-to-female ratio, and immunosuppression, our KTR, LTR, and LngTR groups were each a representative cross-section of their respective organ graft recipient subpopulations within the larger relatively homogenous population of Polish SOTRs. SOT programs in Poland trace back to 1966 (the first KTx), enabling long patient observation periods. This creates a favorable setting for epidemiological analyses of disease risk and prognostic factors, especially in late-onset and extreme late-onset PTLD.

Compared to LTRs, KTRs developed PTLD later, with a greater number of extreme late-onset cases (15–20 years post-transplantation), even after the exclusion of patients who underwent re-transplantation. The presence of EBV in 45–60% of patients in the LTR and KTR groups is in accordance with previous reports of lower EBV positivity in late-onset PTLD [23]. However, these two groups were different in the other factors that affected the time of PTLD onset. Among SOTRs and KTRs, a higher age at the time of first transplantation was associated with earlier PTLD development. Similar effects of patient age have been previously described in KTRs [24,25,26]. However, this correlation was not observed among LTRs, in whom disease onset was affected by treatment with calcineurin inhibitors (CNIs). CsA-treated LTRs developed PTLD significantly earlier than TAC-treated LTRs, although only a small number of LTRs received CsA treatment (2 of 40 patients), which represents a limitation of our study. Notably, the effects of CNIs on PTLD development in the whole SOTR cohort were opposite of those observed in LTRs, supporting our argument against performing analyses in cohorts with mixed graft types. The relatively low number of patients with a history of AR episodes and antibody induction minimizes the interference with our analysis of how maintenance IS agents influenced the time of PTLD onset and patient survival. Relating the IS treatment of our cohort to the IS treatment of the entire population of patients from our centers would provide the best insight. Unfortunately leading to this limitation, we do not possess a unified data system allowing us to trace the IS regimens, doses, and conversions across several thousands of patients in our six transplantation centers. Creating such a database would greatly exceed both the scope and the funding of this study.

All LngTRs in our cohort developed PTLD within the first 12 months post-transplantation, this result is in agreement with reports based on larger European cohorts [27]. Our LngTR cohort exhibited classic early-onset PTLD risk factors; they were significantly younger at transplantation, and 80% had detectable CMV or EBV DNA. In our opinion, this may translate to possible primary EBV infection due to EBV donor/recipient mismatch (a known early-onset PTLD risk factor) as the underlying cause of early disease development in these LngTRs. We were not able to verify this claim, as lung donor EBV serology is not routinely performed in Poland. Another possible cause might stem from the higher intensity of IS, especially within the first year post-transplant, but it seems less likely, as only two out of five patients in our cohort received either IS induction or AR treatment. Unfortunately, due to the limited number of lung transplantations performed in Poland to date, the LngTR cohort was too small to enable informative statistical analysis of risk and prognostic factors.

In all groups, monomorphic B-cell PTLD was the most common disease type. Monomorphic T-cell PTLD was the second most common disease type among KTRs, with a frequency of 12.5% this finding falls within limits reported in the literature [28]. LTRs exhibited a somewhat high frequency of PH-PTLD, which had a negative impact on survival; this finding is not previously described in the literature, and its significance is not yet clear. Most studies with larger cohorts published to date focus on polymorphic and monomorphic B-cell PTLD by design [29]. We consider non-destructive PTLD to be significantly underreported and underrepresented in the literature; studies with similar LTR cohorts produced lower PH-PTLD frequencies [14]. It is possible that these patients harbored more than one histological PTLD subtype, potentially leading to inadequate choice of treatment.

Although IS reduction shows variable efficacy in more histologically advanced or EBV-negative PTLD, this intervention remains the first-line treatment for all forms of PTLD. [30] Within our cohort, it was the most common type of intervention in all groups but was significantly correlated with improved survival only among KTRs. Further analysis of the role of IS reduction in KTRs with PTLD, preferably in a prospective manner and with the adjustment for possible confounders needed to verify the validity of this finding.

Our analysis showed that survival was negatively impacted by prior use of ATG, either as induction or acute rejection treatment. However, this factor was present in only three KTRs and one LTR; therefore, we consider the 95% CIs too wide for meaningful interpretation as a stand-alone result. Additionally, the number of patients who received ATG was too low to attempt stratification of other survival results based on this parameter.

Treatment regimens containing anti-CD20 (either as monotherapy or combined with cyclophosphamide, doxorubicin, vincristine, and prednisone) were found to promote survival among SOTRs. This is in accordance with the results of multicenter PTLD-1 and PTLD-2 clinical trials, showing improved patient survival in monomorphic B-cell PTLD [31,32]. The effect of anti-CD20 was insignificant among KTRs or LTRs alone. As previously reported, focal disease and surgical treatment were also found to promote favorable outcomes among SOTRs [33].

The significantly lower overall survival among KTRs may have been correlated with the longer interval between transplantation and PTLD development in these patients, which could have resulted in a higher burden of IS. The high number of PTLD-related deaths among KTRs may be associated with the higher frequencies of disseminated disease (Lugano stages II-IV) and of PTLD types with lower treatment success rates (monomorphic T-cell PTLD) [34]. Among both KTRs and LTRs, the overall survival was lower than the reported 5- and 10-year survival rates of graft recipients from the centers participating in this study [35,36]. Future verification of these findings will require the analysis of possible confounding comorbidities (e.g., cardiovascular, diabetic, and infectious). Novel technologies including 3D-bioprinting and spheroid lymphoma cultures [37] as well as novel treatment strategies such as CAR-T lymphocytes [38] might allow for a better understanding of PTLDs’ microenvironment and improved patient survival.

We believe that some of the limitations of this study are related to the long observation period of over 20 years. In that time, there have been significant changes in clinical practice and in medical data storage systems. Consequently, data regarding EBV donor/recipient serology and EBER results were not available for many of our patients, which we consider the greatest limitation of this study.

## 5. Conclusions

In summary, here, we analyzed a cohort of SOTRs diagnosed with PTLD and exhibiting a typical pattern of risk and prognostic factors (as per the literature) and demonstrated that the impact of these factors appeared to differ among isolated organ-specific groups. Furthermore, organ-specific groups differed in the factors that affected the time of PTLD onset and patient survival. There remains a need for studies in larger multicenter cohorts that will enable the analysis of risk and prognostic factors and outcomes in an organ-specific fashion. Such an approach is more likely to result in the development of patient-tailored clinical tools, allowing transplant physicians to better identify patients at risk and predict treatment outcomes.

## Figures and Tables

**Table 1:** Patient characteristics.

	KTRs	LTRs	LngTRs	
**PATIENT CHARACTERISTICS**	**n (%)**			***p*-Value**
Female	20 (34.5)	13 (32.5)	3 (60.0)	0.474
Male	38 (65.5)	27 (67.5)	2 (40.0)	0.474
Median age at PTLD Dx, years	49	50	25	0.005
Median age at first Tx, years	40	45	24	0.009
Median time from Tx to PTLD Dx, months	117	49	5	<0.001
Re-transplantation	3 (5.2)	3 (7.5)	1 (20.0)	0.438
**IS TREATMENT**				
**Induction treatment**				
ATG induction	3 (7.5)	0 (0.0)	0 (0.0)	0.31
Anti-CD25 induction	8 (14.5)	21 (60.0)	1 (20.0)	<0.001
**Acute rejection treatment**				
ATG-treated AR	0 (0.0)	1 (2.8)	0 (0.0)	0.468
Glucocorticoid-treated AR	15 (31.2)	13 (33.3)	1 (20.0)	0.832
**Maintenance IS**				
Monotherapy	0 (0.0)	7 (17.5)	0 (0.0)	0.003
Double-drug IS	9 (15.5)	14 (40.0)	2 (40.0)	0.02
Triple-drug IS	49 (84.5)	17 (42.5)	3 (60.0)	<0.001
Glucocorticoids	54 (93.1)	24 (60.0)	5 (100.0)	<0.001
Cyclosporin A	35 (60.3)	2 (5.0)	2 (40.0)	<0.001
Tacrolimus	20 (34.5)	38 (95.0)	3 (60.0)	<0.001
Azathioprine	18 (31.0)	6 (15.0)	0 (0.0)	0.082
Mycophenolate	37 (63.8)	16 (40.0)	1 (20.0)	0.022
Sirolimus	0 (0.0)	0 (0.0)	2 (40.0)	<0.001
Everolimus	3 (5.2)	0 (0.0)	0 (0.0)	0.302
**VIRAL INFECTIONS**				
EBV positivity	19 (45.2)	19 (59.4)	4 (80.0)	0.223
CMV DNA	4 (8.7)	2 (5.7)	4 (80.0)	<0.001
HBV DNA	5 (10.4)	3 (8.1)	0 (0.0)	0.721
HCV RNA	6 (13.0)	8 (21.1)	0 (0.0)	0.369

AR, acute rejection; ATG, anti-thymocyte globulin; Dx, diagnosis; EBV positivity, as described in the Materials and Methods section (Epstein–Barr virus DNA, LMP1, or EBER ISH); Tx, transplantation. *p*-values represent the chi-squared and Kruskal–Wallis (for median time parameters) test comparison between KTRs, LTRs, and LngTRs.

**Table 2:** Factors affecting the time of PTLD onset—univariate analysis.

Parameter	HR	95% CI	*p*-Value
Total SOTRs			
Age at 1st Tx	1.02	1.00–1.04	0.01
Tacrolimus	1.96	1.3–2.95	0.001
Cyclosporin A	0.63	0.42–0.94	0.025
KTRs			
Age at 1st Tx	1.03	1.00–1.05	0.01
LTRs			
Tacrolimus	0.21	0.05–0.95	0.042
Cyclosporin A	4.8	1.06–21.78	0.042

PTLD, post-transplantation lymphoproliferative disorder; HR, hazard ratio; CI, confidence interval; SOTR, solid organ transplant recipient; Tx, transplantation. *p*-values for univariate Cox regression.

**Table 3:** Factors affecting the time of PTLD onset—multivariate analysis.

Parameter	HR	95% CI	*p*-Value
Total SOTRs			
Age at 1st Tx	1.03	1.01–1.04	0.006
Tacrolimus	2.02	0.94–4.34	0.07
Cyclosporin A	1.03	0.46–2.3	0.94
EBV DNA	1.36	0.85–2.18	0.20
KTRs			
Age at 1st Tx	1.04	1.01–1.07	0.003
Tacrolimus	1.45	0.55–3.8	0.45
Cyclosporin A	0.92	0.35–2.43	0.87
EBV DNA	1.24	0.63–2.41	0.53
LTRs			
Age at 1st Tx	1.03	0.99–1.07	0.06
Tacrolimus	0.27	0.06–1.28	0.10
EBV DNA	1.33	0.61–2.91	0.48

PTLD, post-transplantation lymphoproliferative disorder; HR, hazard ratio; CI, confidence interval; SOTR, solid organ transplant recipient; Tx, transplantation. *p*-values for multivariate Cox regression.

**Table 4:** PTLD characteristics.

	KTRs	LTRs	LngTRs	
**PTLD Characteristics**	**n (%)**			***p*-Value**
Lugano stage I	24 (42.1)	20 (50.0)	2 (40.0)	0.724
Lugano stages II–IV	33 (57.9)	20 (50.0)	3 (60.0)	0.724
Early onset	5 (8.6)	7 (17.5)	5 (100.0)	<0.001
Late onset	53 (91.4)	33 (82.5)	0 (0.0)	<0.001
Post-mortem diagnosis	1 (1.7)	3 (7.5)	0 (0.0)	0.312
ICC 2022 type				
Nondestructive				
Florid follicular hyperplasia	0 (0.0)	1 (2.5)	0 (0.0)	0.451
Infectious mononucleosis	1 (1.7)	2 (5.0)	1 (20.0)	0.114
Plasmacytic hyperplasia	1 (1.7)	6 (15.0)	0 (0.0)	0.031
Polymorphic	5 (8.6)	6 (15.0)	0 (0.0)	0.441
Monomorphic B-cell	36 (64.3)	23 (57.5)	4 (80.0)	0.562
Monomorphic T-cell	7 (12.5)	1 (2.5)	0 (0.0)	0.161
Classic Hodgkin lymphoma	6 (10.7)	1 (2.5)	0 (0.0)	0.243

PTLD, post-transplantation lymphoproliferative disorder; KTRs, kidney transplant recipients; LngTRs, lung transplant recipients; LTRs, liver transplant recipients. Early onset is <12 months after transplantation, and late onset is >12 months after transplantation. *p*-values represent the chi-squared test comparison between KTRs, LTRs, and LngTRs.

**Table 5:** PTLD treatment and outcomes.

	KTRs	LTRs	LngTRs	
**PTLD Treatment**	**n (%)**			***p*-Value**
Reduction of IS	49 (84.5)	34 (87.2)	4 (80.0)	0.881
Conversion to Sirolimus	13 (22.4)	10 (25.6)	1 (20.0)	0.918
Conversion to Everolimus	5 (8.6)	2 (5.1)	0 (0.0)	0.66
Anti-CD20	14 (24.6)	13 (33.3)	3 (60.0)	0.205
R-CHOP	8 (14.0)	16 (41.0)	1 (20.0)	0.01
CHOP	6 (10.7)	5 (12.8)	0 (0.0)	0.686
Other chemotherapy	19 (33.9)	7 (18.4)	0 (0.0)	0.096
Surgery	15 (25.9)	12 (30.8)	2 (40.0)	0.733
Radiotherapy	9 (15.5)	2 (5.0)	0 (0.0)	0.185
Outcomes				
CR	27 (46.6)	24 (60.0)	4 (80.0)	0.2
Total deaths	29 (50.0)	11 (27.5)	1 (20.0)	0.053
PTLD-related deaths	25 (43.1)	7 (17.5)	1 (20.0)	0.024
Alive	17 (29.3)	24 (60.0)	4 (80.0)	0.003
Lost to follow-up	13 (22.8)	5 (12.5)	0 (0.0)	0.241

PTLD, post-transplantation lymphoproliferative disorder; KTRs, kidney transplant recipients; LngTRs, lung transplant recipients; LTRs, liver transplant recipients; CR, complete remission; IS, immunosuppression; R-CHOP, anti-CD20 + CHOP. *p*-values represent the chi-squared test comparison between KTRs, LTRs, and LngTRs.

**Table 6:** Factors affecting patient survival—univariate analysis.

Parameter	HR	95% CI	*p*-Value
Total SOTRs			
Lugano stage I	0.36	0.17–0.73	0.005
Lugano stages II–IV	2.89	1.42–5.88	0.004
Surgery	0.33	0.14–0.80	0.014
Anti-CD20	0.43	0.19–0.98	0.045
R-CHOP	0.39	0.15–0.99	0.047
ATG induction	12.53	3.53–44.45	<0.001
AR ATG	28.83	3.0–277.17	0.004
KTRs			
ATG induction	11.93	3.04–46.83	<0.001
Lugano stage I	0.41	0.17–1.0	0.049
Lugano stages II–IV	2.51	1.04–6.02	0.04
Reduction of IS	0.34	0.14–0.82	0.017
LTRs			
AR ATG	34.5	2.16–551.55	0.012
PH PTLD	4.24	1.23–14.6	0.022

AR, acute rejection; ATG, anti-thymocyte globulin; PH, plasmacytic hyperplasia; R-CHOP, anti-CD20 + cyclophosphamide, doxorubicin, vincristine, and prednisone. *p*-values for univariate Cox regression.

**Table 7:** Factors affecting patient survival—multivariate analysis.

Parameter	HR	95% CI	*p*-Value
Total SOTRs			
Age at 1st Tx	1.03	1.00–1.06	0.032
Lugano I	0.44	0.18–1.07	0.07
Surgery	0.36	0.13–1.02	0.054
IR	0.59	0.2–1.76	0.34
EBV DNA	0.87	0.41–1.83	0.72
KTRs			
Age at 1st Tx	1.03	1.00–1.07	0.045
Lugano I	0.38	0.12–1.22	0.10
IR	0.36	0.09–1.35	0.13
EBV DNA	0.73	0.27–1.95	0.53
**LTRs**			
Age at 1st Tx	1.01	0.95–1.07	0.74
Lugano I	0.43	0.10–1.81	0.25
Plasmacytic hyperplasia	10.2	2.2–47.48	0.003
EBV DNA	3.08	0.51–18.45	0.22

PTLD, post-transplantation lymphoproliferative disorder; HR, hazard ratio; CI, confidence interval; SOTR, solid organ transplant recipient; Tx, transplantation. *p*-values for multivariate Cox regression.

## Data Availability

The data that support the findings of this study are available from the corresponding author upon reasonable request.
